# Formica-rufa

**Published:** 1896-01

**Authors:** J. W. Thomson

**Affiliations:** New York


### FORMICA-RUFA.

J. W. THOMSON, M. D., NEW YORK.

A gentleman, born in 1810, whom I have treated off and on for the past thirteen years, and whom I saw through an attack of dysentery about ten years ago, had severe neuralgic pains between posterior of right ear and centre of occiput about two years ago. From this he apparently recovered, and I lost sight of him for over a year. Some six months ago he returned to me with a steady sore and burning pain in same region; swollen and tender around, but especially behind, right ear. He can only sleep in just such a comfortable position, which he cannot describe, but only knows when he gets there; and must have it covered up warmly behind right ear and between there and occiput and neck—all right side.

It is always grateful for him to take right hand and rub gently behind right ear, which he often does.

Sweat, while not ungrateful, never affords any relief. Says it neither makes him better nor worse. His whole head seems to sympathize with the distress. The head and front of offending is, however, in the parts described.

He has dribbling of urine, which is a trouble and source of great annoyance.

I gave Formica-rufa, six doses, three per diem (*i. e.*, one before each meal).

On the second day, toward evening, he felt worse, and on the third day there were twinges and spasms of severe pain in lieu of the soreness only he had before experienced, which gradually got better, and on the fourth day even the soreness was much better. The main thing, however, was that he felt mentally better; said his mind and head felt stronger, and from feeling depressed he had become cheerful, and life did not seem the burden it had for some time past. Thus, the God-given law—Homœopathy, and Homœopathy alone, in diseased states—answers affirmatively the question asked by Shakespeare, "Cans't thou minister to a mind diseased?"

